# Improving Quantitative Traits in Self-Pollinated Crops Using Simulation-Based Selection With Minimal Crossing

**DOI:** 10.3389/fpls.2021.729645

**Published:** 2021-09-01

**Authors:** Daisuke Sekine, Mai Tsuda, Shiori Yabe, Takehiko Shimizu, Kayo Machita, Masayasu Saruta, Tetsuya Yamada, Masao Ishimoto, Hiroyoshi Iwata, Akito Kaga

**Affiliations:** ^1^Institute of Vegetable and Floriculture Science, National Agriculture and Food Research Organization, Tsu, Japan; ^2^Institute of Crop Science, National Agriculture and Food Research Organization, Tsukuba, Japan; ^3^Tsukuba Plant Innovation Research Center, University of Tsukuba, Tsukuba, Japan; ^4^Department of Agricultural and Environmental Biology, Graduate School of Agricultural and Life Sciences, The University of Tokyo, Bunkyo-ku, Japan

**Keywords:** seed protein content, soybean, quantitative trait, genomic selection, genomic prediction, segregation simulation, marker-assisted recurrent selection

## Abstract

Genomic selection and marker-assisted recurrent selection have been applied to improve quantitative traits in many cross-pollinated crops. However, such selection is not feasible in self-pollinated crops owing to laborious crossing procedures. In this study, we developed a simulation-based selection strategy that makes use of a trait prediction model based on genomic information to predict the phenotype of the progeny for all possible crossing combinations. These predictions are then used to select the best cross combinations for the selection of the given trait. In our simulated experiment, using a biparental initial population with a heritability set to 0.3, 0.6, or 1.0 and the number of quantitative trait loci set to 30 or 100, the genetic gain of the proposed strategy was higher or equal to that of conventional recurrent selection method in the early selection cycles, although the number of cross combinations of the proposed strategy was considerably reduced in each cycle. Moreover, this strategy was demonstrated to increase or decrease seed protein content in soybean recombinant inbred lines using SNP markers. Information on 29 genomic regions associated with seed protein content was used to construct the prediction model and conduct simulation. After two selection cycles, the selected progeny had significantly higher or lower seed protein contents than those from the initial population. These results suggest that our strategy is effective in obtaining superior progeny over a short period with minimal crossing and has the potential to efficiently improve the target quantitative traits in self-pollinated crops.

## Introduction

Plant breeding has played a crucial role in the development of human societies. The demands of the increasing world population could be met by improving yield and nutrient content in self-pollinating crops such as rice, wheat, and soybean, which account for a large part of the human food supply (FAO, 2015), through breeding better varieties (Tester and Langridge, 2010). Conventional breeding programs for self-pollinated crops typically use the bulk population method, where a segregating population is generated and repeatedly selfed over several generations without selection, followed by the selection of genetically fixed plants with favorable traits (Brown and Caligari, 2008). Important agronomic traits such as yield and nutrient content are known to be controlled by multiple genes, which are defined as quantitative trait loci (QTLs). Efficient selection of plants with multiple favorable QTLs from a segregating population can be challenging and requires a large population size. Such plants can be obtained from a typical breeding population through repeated selection, followed by crossing between selected individuals known as a recurrent selection strategy (John, 1987). However, this selection strategy is not very efficient in improving target quantitative traits due to the low selection accuracy based on the phenotype of a single plant (Bos and Caligari, 2008). Molecular markers can help to improve the selection of a target trait (Bernardo, 2008), and they are used for the introgression of specific favorable alleles, such as in marker-assisted gene pyramiding, marker-assisted recurrent selection (MARS), and genomic selection (GS) (Lande and Thompson, 1990; Meuwissen et al., 2001; Bernardo, 2008). MARS allows the accumulation of a relatively large number of medium-effect QTLs by using a subset of markers that are significantly associated with target traits, whereas GS increases the total additive genetic effect, including small-effect QTLs, using genome-wide markers. These approaches aim to increase the frequency of multiple favored QTLs in a population (Bernardo, 2008).

Several empirical studies in cross-pollinated crops have shown that MARS and GS can improve quantitative target traits over conventional phenotypic selection (Eathington et al., 2007; Massman et al., 2013; Beyene et al., 2015, 2016; Yabe et al., 2018). However, GS and MARS are not easy in self-pollinated crops of barley and soybean, as it is difficult to obtain a sufficient number of F_1_ hybrid seeds for use in the subsequent generation (Bernardo, 2010). For example, in soybean, only two or three F_1_ hybrid seeds are obtained per hand-crossing event. To better apply GS and MARS to self-pollinated crops, Bernardo (2010) proposed that selection and crossing should be performed primarily on the F_2_ generation, which is by selfing of a few F_1_ seeds. It was determined that genetic improvement is only slightly lower using this method than that using the F_1_ generation (Bernardo, 2010). However, this method requires the production of F_1_ hybrid seeds from multiple cross combinations between selected plants, which is not easily implemented in most self-pollinated crops, where crossing by hand is laborious. For instance, in soybean, the floral organs are very small and emasculation must be conducted prior to flowering. Moreover, since abscission occurs in 20–80% of the flowers and pods at any stage of soybean seed development (Candwell, 1973), repeated hand crossing is necessary to obtain hybrid seeds in each cross combination. Thus, to apply GS and MARS to many self-pollinated crops, a strategy for reducing the labor of hand crossing is needed.

Here, we describe the identification of the best cross combinations; thus, enabling us to improve a target trait with minimal hand crossing. The usefulness criterion (*U* = μ+*iσ*_*g*_*h*) and superior progeny values (*s* = μ+*iσ*_*g*_), where μ is the expected cross mean trait value, *i* is the standardized selection intensity, σ_*g*_ is the genetic standard deviation of the cross, and *h* is the square root of trait heritability, have been proposed as a selection criteria for cross combinations (Schnell and Utz, 1975; Zhong and Jannink, 2007). However, it is difficult to estimate the progeny variance ($\sigma_{g}^{2}$) for each cross combination prior to crossing. Lehermeier et al. (2017) proposed an analytical approach based on the whole-genome regression model given in the training population to predict the progeny variance of each cross combination. Separately, Iwata et al. (2013) proposed a method for predicting trait variance in the progeny population immediately after training the population by using simulated progeny genotypes and a whole-genome regression model. Similarly, Mohsen et al. (2015) developed the computing package PopVar, which can predict progeny variance in each cross combination. PopVar is assumed to predict the superior progeny values for each cross when the progeny genotypes are fixed, such as in recombinant inbred lines (RILs). The method proposed by Iwata et al. (2013) would be useful for GS and MARS, as selection and crossing are repeated in each cycle. We sought to develop an efficient selection strategy that can quickly produce superior progeny with a minimal crossing in self-pollinated crops by combining the concepts proposed by Bernardo (2010) and Iwata et al. (2013). The efficiency of our strategy was evaluated based on simulated and empirical experiments leveraging a soybean breeding population.

## Materials and Methods

### Experimental Design for the Simulation Study

The plant species was assumed to be diploid with 20 pairs of chromosomes (*n* = 20), each with a length of 150 cm. A trait was considered to be controlled by 30 or 100 QTLs. The QTLs were assumed to be randomly distributed among the 20 chromosome pairs. The additive effect of each QTL was sampled independently from a gamma distribution whose scale and shape parameters were 1.66, and 0.4, respectively, as described by Meuwissen et al. (2001). The genetic variance of the initial population was set to 1.0. The heritability (*h*^2^) of the trait was assumed to be 0.3, 0.6, or 1.0. Genetic variation was explained only by an additive effect, and no dominance or epistatic effects influenced the traits. All QTLs were biallelic, and each genotype was defined as 1 (AA), 0 (AB), or −1 (BB). One F_1_ genotype, heterozygous for all QTL alleles, was selfed to produce the initial F_2_ population. Selfing was repeated by single-seed descent procedure up to the F_8_ generation, which was then used as an initial population for the simulation study. The population size was set to 200 plants. The genetic value (GV) of each plant was calculated as the sum of the true QTL effect multiplied by its genotype. The environmental effect of each plant was sampled independently from a normal distribution, and the phenotypic value for each plant was calculated as the sum of GVs and environmental effects. The prediction model was constructed using the following linear regression model:

$$\begin{array}{l} y_{i}=\mu +\overset{m}{\underset{j=1}{\sum }}\beta_{j}x_{ij}+\varepsilon_{i} \end{array}$$

where *y*_*i*_ is the phenotypic value of plant *i* (*i* = 1, 2, …., *n*), μ is the overall mean, and β_*j*_ and ε_*i*_ represent the genetic effect coefficients of QTL *j* (*j* = 1, 2,., *m*). The error deviation is assumed to follow N(0, $\sigma_{\text{ε}}^{2}$). In addition, *x*_*ij*_ denotes the genotype of QTL, *j* for line and *i* for individual. The least absolute shrinkage and selection operator (LASSO) method from glmnet Version 2.0-18 (Friedman et al., 2010), in which selection of a variable and estimation of the genetic effect coefficients are simultaneously conducted, was used to estimate the genetic effect coefficient and calculate the prediction value (PV) in R version 3.4.1 (R Core Team, 2018). The penalty parameter (λ_L_) was optimized based on a ten-fold cross-validation method.

**Figure 1:**
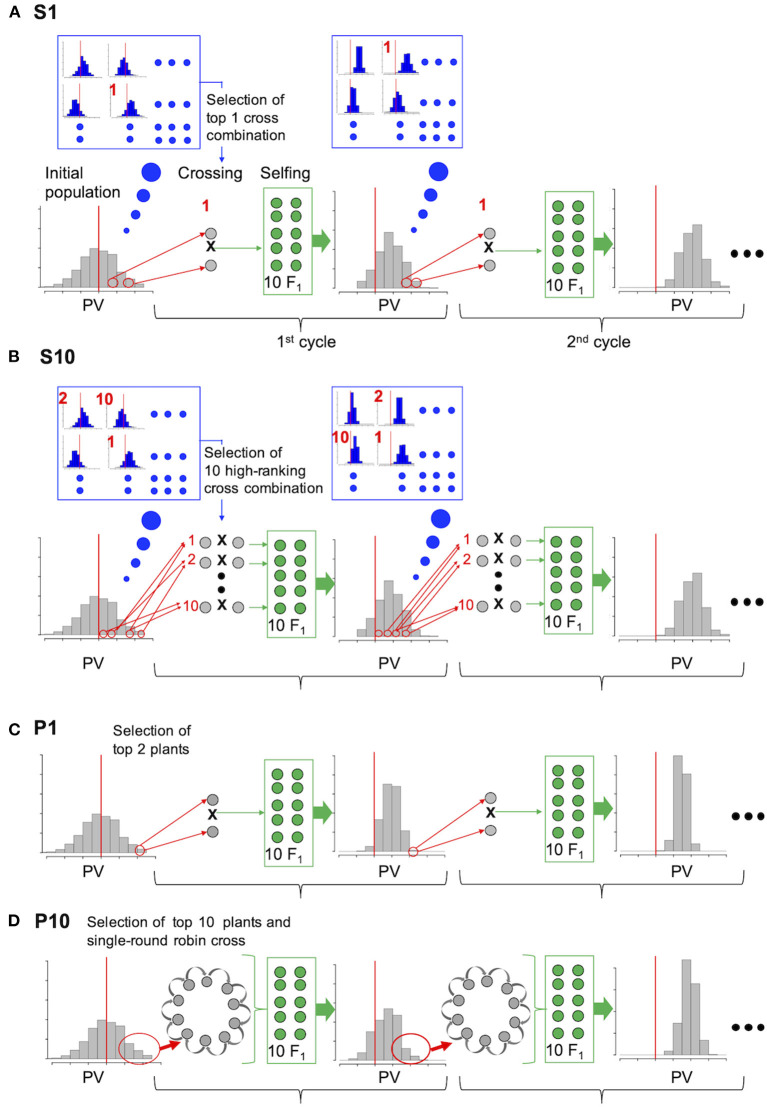
Comparison of the selection strategies based on simulation experiment. **(A)** and **(B)** are the proposed strategies (S1, S10); genotypes of the initial population were used to simulate the distribution of prediction values (PVs) in the F_2_ progeny (blue enlarged frame) for all possible cross combinations. Cross combinations are ranked according to the mean PV of the top 10 progeny, and crosses of only the top combination (**A**, S1) or top 10 combinations (**B**, S10) were conducted in each cycle. **(C)** P1: Top 1 and 2 plants were selected on the basis of their own PV and crossed in each cycle. **(D)** P10: Top 10 plants were selected on the basis of their own PV and crossed in a single-round robin design in each cycle.

By using the initial population, we compared the selection efficiency of the two major different strategies to select parents for crossing based on prediction values of population (P) and simulated prediction values of the progenies (S) (Figure 1). The selection scheme (used for all strategies) was as follows: F_2_ progeny (treated as a single population) was generated from 10 F_1_ plants. All selection and crossing procedures for producing the next generation were performed using the F_2_ population in each cycle.

For selection strategy S1 (simulated prediction value; one cross combination), the two plants with the best performance among all possible cross combinations were selected on the basis of the simulated PV of their progeny and crossed. For selection strategy S10 (simulated prediction value; 10 cross combinations), the top 10 cross combinations were selected and crossed. For selection strategy P1 (prediction value; one cross combination), the top one and two plants from the initial population were selected on the basis of their own PV and crossed to generate progeny in each cycle. For selection strategy P10 (prediction value: 10 cross combinations), the top 10 plants from the initial population were selected on the basis of their own PVs and crossed using a single-round robin design, in which crossing was conducted for 10 cross combinations as a chain, for example, plant1 × plant2, plant2 × plant3,…, plant10 × plant1, to generate progeny in each cycle. Selection strategy P10 is a substitute for that proposed by Bernardo (2010) where all possible cross of selected plants was assumed.

In the present study, two more intermediate conditions were examined, which are P5 (prediction value; five cross combinations) and S5 (simulated prediction value; five cross combinations). To adjust the 10 F_1_ genotypes generated in each selection strategy, 10 F_1_ genotypes were generated from the selected cross combination in S1 and P1, two from each of the five cross combinations in S5 and P5, and one from the 10 cross combinations in S10 and P10. An equal number of F_2_ genotypes were generated by selfing each F_1_ plant. The population size of the F_2_ plants was fixed at 200.

In S1, S5, and S10, F_2_ genotypes were simulated for all possible cross combinations, and the PV of each F_2_ plant was then calculated using its simulated genotype and the prediction model. Cross combinations were ranked according to the mean PVs of the top 10 F_2_ plants in each population. Only one top cross combination was selected in strategy S1, whereas the top five and 10 cross combinations were selected to generate the next progeny in strategies S5 and S10, respectively. Unlike strategies S1, S5, and S10, each plant within a population was ranked according to its own PV in the same manner as strategies P1, P5, and P10.

The selections were continued up to the fifth cycle, updating the prediction model at odd numbered cycles. In even numbered cycles the prediction model built in the previous cycle was used. In the present study, 50 simulations were performed independently, and the mean and variance of the simulation replications were reported. Improvement of genetic gain was evaluated using maximum GV (MGV), which was calculated for plants in each F_2_ population in every cycle. A matched paired *t*-test using the MGVs of each F_2_ population from 50 simulation replications was used to examine the significance of the difference in the improvement of GVs between strategies. The *P-*value was adjusted using the Bonferroni method (Bonferroni, 1936). In the *t-*test, populations derived from identical initial populations in each replication were considered match-pairs. The changing patterns of genetic variation during selection cycles were evaluated based on the proportion of fixed favorable and unfavorable QTL alleles and that of unfixed QTL alleles within populations. Selection accuracy was evaluated using Pearson's correlation coefficient between PVs and GVs in each cycle. In some simulation replications, the QTL genotypes in the population were completely fixed in a particular cycle. In this case, the data of the last cycle were continuously used until the end of the selection period. All the simulation scripts were written and run in R version 3.4.1 (R Core Team, 2018) using the Breeding Scheme Language package (Yabe et al., 2017).

### Plant Materials and Growth Conditions

Seeds were obtained from the National Agriculture and Food Research Organization (NARO) at Genebank, Japan. A cross between two the varieties of soybean (*Glycine max*), “Enrei” (JP 28862) as the female parent and “Hyuga” (JP 29640) as the male parent, was performed to produce the F_2_ population. Selfing was repeated by a single-seed descent procedure up to the F_8_ generation, generating a population consisting of 194 lines, which were genotyped. RILs from F_8_ and later generations were grown at four separate locations; the details of the growing conditions are summarized in Supplementary Table 1.

### Crosses and Development of Lines for the Validation of Selection Effect

Breeding lines were developed using two-cycle crosses. First the RILs were crossed and then their progenies were crossed. In the first cycle, crosses between selected RILs were performed following proposed strategy S1, and several F_1_ seeds (defined as the first F_1_) were obtained in 2014. The first F_1_ plants were grown in a greenhouse in 2014 and F_2_ seeds (defined as the first F_2_) were sown on June 19, 2015. In the second cycle, crosses between selected first F_2_ plants were performed, and several F_1_ seeds (defined as the second F_1_) were collected. The second F_1_ plants were grown in a greenhouse in 2015 and F_2_ seeds (defined as the second F_2_) were sown on June 20, 2016, and F_3_ seeds (defined as the second F_3_) were obtained through self-pollination. The second F_3_ generation was used for validation, and these seeds were sown on July 4, 2017. These validation lines, parental cultivars, and selected RILs were grown in a randomized complete block design with eight replications, all under the same growth conditions (Supplementary Table 1).

### Analysis of Trait Data

Seed protein content was measured for the RILs, parental cultivars, and second F_3_ plants using near-infrared spectroscopy with an Infratec 1241 Grain Analyzer (calibration model SO138111 Soybean STM, FOSS North America, Eden Prairie, MN, USA). Transmission spectra were recorded in the wavelength range of 570–1,100 nm. As for validation of selection effect on the protein content data set obtained in 2017, statistically significant differences between blocks were analyzed using a one-way ANOVA followed by a Tukey–Kramer *post hoc* test. All statistical analyses were conducted using R version 3.4.1 (R Core Team, 2018).

### SNP Marker Analysis

As described previously (Khosla et al., 1999), total genomic DNA was extracted from young fresh leaves (3 g) taken from the parental cultivars, RILs, first F_1_, first F_2_, second F_1_, and second F_2_ plants using guanidine hydrochloride (Sigma-Aldrich) and proteinase K (QIAGEN). Multiplex assays for 513 SNPs, distributed throughout the genome, were designed (Supplementary Table 2) using Sequenom Assay Design 3.1 (Sequenom). Genotyping was conducted using the Sequenom MassARRAY system (Oeth et al., 2009). Multiplex PCR, followed by template-directed single-base extension at each SNP, was conducted using the MassARRAY iPLEX Gold kit (Sequenom), following the instructions of the manufacturer. Genotypes were determined using MassARRAY Typer version 4.0 (Sequenom).

### Linkage Map Construction and QTL Detection

The linkage map of RILs was constructed using JoinMap v. 4.0 (Van Ooijen and Voorrips, 2001). The logarithm of the odds threshold for the grouping of DNA markers ranged from 3.0 to 5.0. The marker order was determined using the maximum-likelihood mapping algorithm. The recombination frequency was converted into the genetic distance (cM) using the Haldane mapping function. QTL analysis was conducted using Multi-QTL ver. 2.6 (Multi-QTL, http://www.multiqtl.com). Seven phenotypic datasets for the RILs, taken from 2009 to 2013, were used to perform multienvironment multiple interval mapping (ME-MIM) to scan the entire genome (Korol et al., 1998, 2001). Statistical significance thresholds (α = 0.05) for the identification of putative QTLs were tested by permutation with 10,000 runs (Churchill and Doerge, 1994), following which the parameters of significant QTLs were reported as position, additive effects, and percentage of variance explained.

### Genome-Wide Association Analysis

A variational method for Bayesian hierarchical regression (VBAY) model, from the PUMA package (Hoffman et al., 2013), was used for the detection of markers associated with seed protein content. VBAY uses a Bayesian framework and, hence, reports the posterior probability when each marker coefficient is nonzero. This can be interpreted as the posterior probability that the marker is significantly associated with the phenotype. A posterior probability value of >0.5 was used as the significance threshold for calling associations with the phenotype.

### Construction of the Prediction Model

Multienvironment multiple interval mapping detects relatively robust markers associated with a target trait, whereas VBAY can identify weak associations at a low false-positive rate (Logsdon et al., 2010). The representative 29 markers with high genotyping quality, located in the genomic region detected by either of the methods, were used as variables in the prediction model for protein content. In addition to the simulation experiment, LASSO, from the R package glmnet Version 2.0-18 (Friedman et al., 2010) was used to construct the prediction model. The penalty parameter (λ_L_) was optimized based on a 10-fold cross-validation method. The accuracy of the model constructed on the basis of the selected marker set and all available markers was compared by implementing 10-fold cross-validation.

## Results

### Selection Efficiency

**Figure 2:**
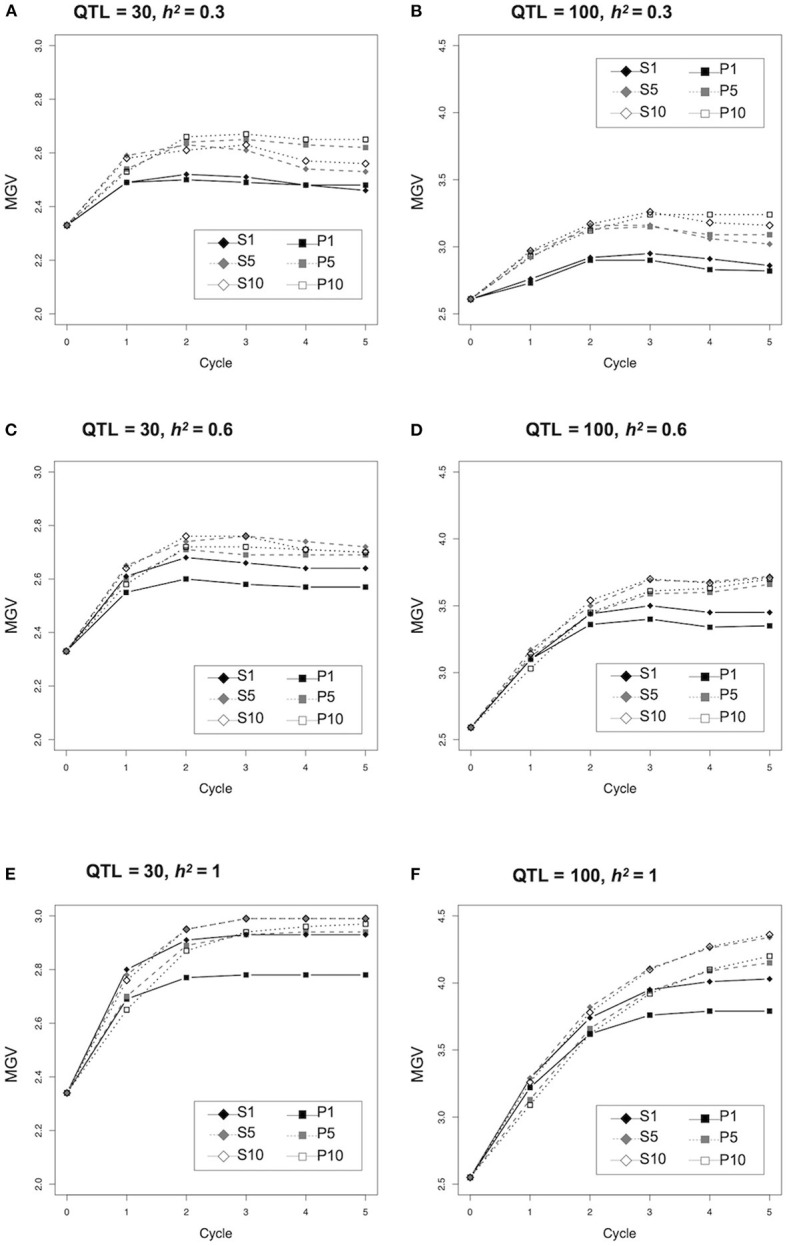
Comparison of genetic improvement during selection cycle of the different selection strategies. Y-axis indicates the maximum genetic values (MGVs).

We assessed the efficiency with which genetic improvement occurred in the two major strategies used to select parents for crossing based on prediction values of the population (P) and simulated prediction values of the progeny (S) (Figure 1). The population MGVs under different conditions, number of QTLs (*n* = 30 or 100) and heritability (*h*^2^ = 0.3, 0.6, or 1.0) were compared (Figure 2; Supplementary Tables 3–5). No difference was observed between the MGVs of S1 (simulated prediction value; 1 cross combination) and P1 (prediction value; 1 cross combination) when QTL = 30 and *h*^2^ = 0.3 (Figure 2A), whereas S1 was significantly higher than P1 when QTL = 30 and *h*^2^ = 0.6 or 1.0 (Figures 2C,E; Supplementary Tables 3, 4). MGVs for the single cross strategies (S1 and P1) were significantly lower than in the multiple cross strategies, i.e., S5, S10 (simulated prediction value; five or 10 cross combinations), and P5, P10 (prediction value; five or 10 cross combinations) during selection cycles when QTL = 30 and *h*^2^ = 0.3 (Figure 2A; Supplementary Tables 3, 4), while no significant difference was observed between S1 and any of the multiple crosses strategies based on prediction values P5 or P10 when QTL = 30 and *h*^2^ = 0.6 or 1.0 (Figures 2C,E; Supplementary Tables 3, 4), except for the difference between S1 and P10 when QTL = 30 and *h*^2^ = 1.0 in later cycles (Figure 2E; Supplementary Tables 3, 4). Although differences between the multiple cross strategies (S5 versus S10 and P5 versus P10) were small when QTL = 30 or 100 and *h*^2^ = 0.3 or 0.6 (Figures 2A–D; Supplementary Tables 4, 5), the MGVs for S5 and S10 were significantly higher than those of P5 and P10 when QTL = 30 or 100 and *h*^2^ = 1.0 (Figures 2E,F; Supplementary Tables 4, 5). Overall, genetic improvement in all selection strategies plateaued by the third cycle, except when QTL = 100 and *h*^2^ = 1.0 (Figure 2).

**Figure 3:**
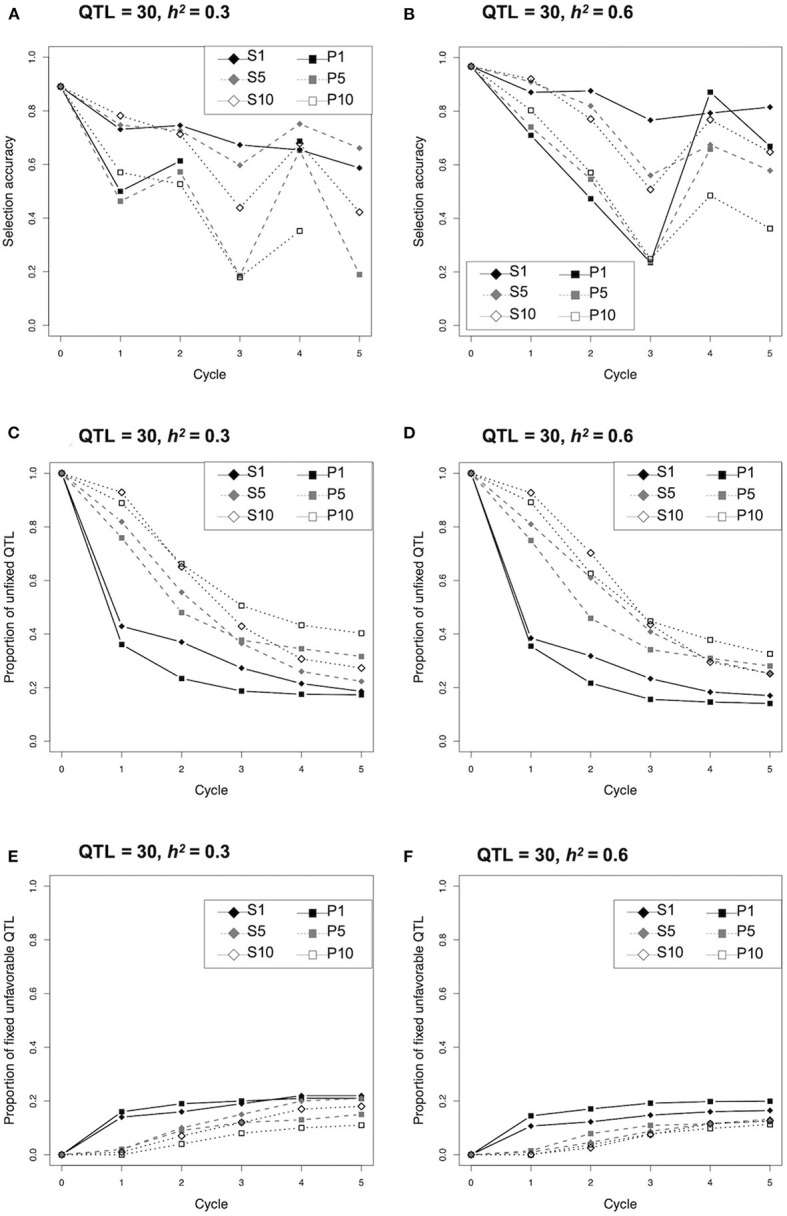
Comparison of selection accuracy, proportion of unfixed quantitative trait loci (QTLs), and fixed unfavorable QTLs during selection cycle of the different selection strategies under the condition of 30 QTLs and *h*^2^ = 0.3 or 0.6.

Since selection strategy S1 revealed a clear response to the heritability as described above (Figure 2), the changing pattern of selection accuracy and proportion of unfixed and fixed unfavorable QTLs were compared between the two different conditions (i.e., 30 QTLs and *h*^2^ = 0.3, or 0.6) (Figure 3; Supplementary Tables 6, 7). The selection accuracy of all strategies at *h*^2^ = 0.3 were less than that at *h*^2^ = 0.6 (Figures 3A,B; Supplementary Table 6). The remarkable difference between *h*^2^ = 0.3 and 0.6 was not observed for the proportion of unfixed and fixed unfavorable QTLs (Figures 3C–F; Supplementary Table 7). The proportion of unfixed QTLs in both the single cross strategies (S1 and P1) dropped rapidly during the early cycles and remained at a low level, while those of multiple cross strategies (i.e., S5, S10, P5, P10) gradually decreased during the selection (Figures 3C,D; Supplementary Table 7). In contrast, the proportion of fixed unfavorable QTLs for both the single cross strategies (S1 and P1) rapidly increased during the first cycle, whereas those of multiple cross strategies gradually increased (Figures 3E,F; Supplementary Table 7).

### Marker Selection and Prediction Model Construction

**Figure 4:**
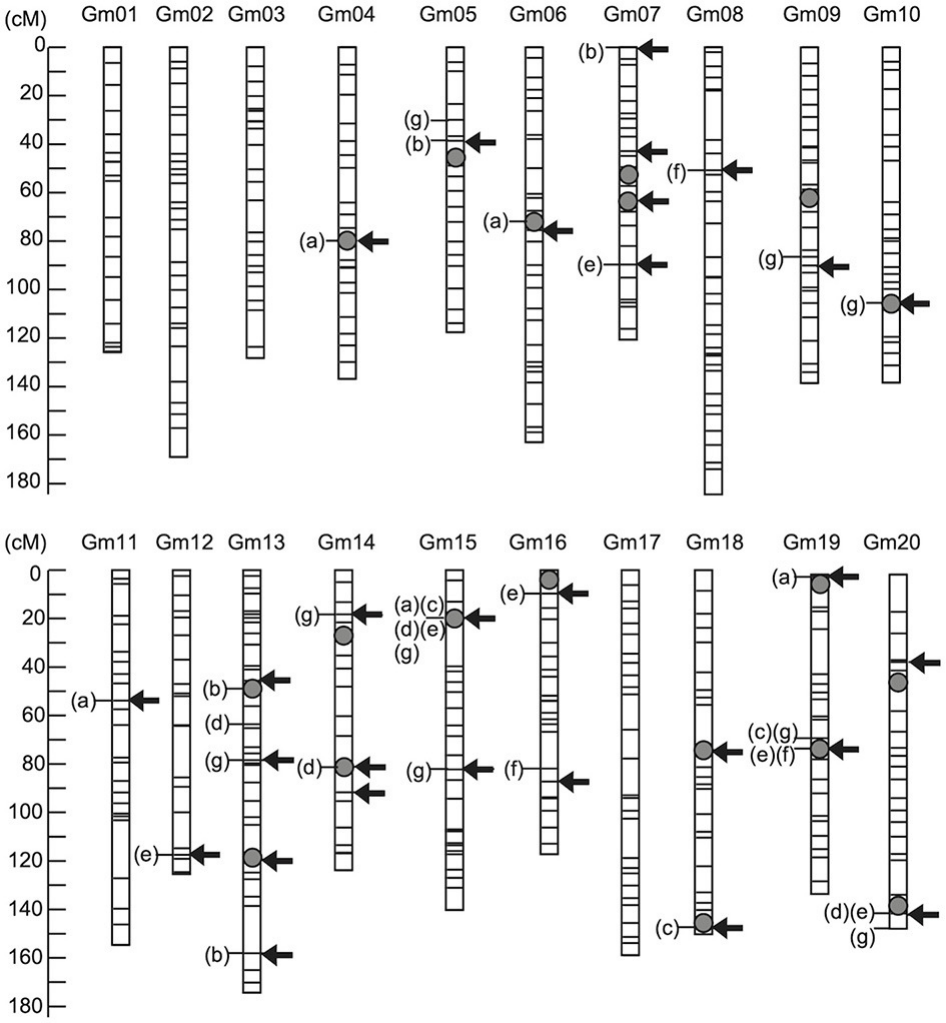
Genetic map locations of genetic factors controlling seed protein content. The arrows indicate marker positions used to construct the prediction model of protein content. The grey circle indicates the quantitative trait locus (QTL) positions detected using multienvironment multiple interval mapping (ME-MIM) analysis. The letters in parentheses show the name of yearly data set for which significant association of the marker with protein content was detected using a variational method for Bayesian hierarchical regression (VBAY): (a) NICS1-2009, (b) NICS2-2010, (c) WARC-2011, (d) WARC-2012, (e) NICS3-2012, (f) NICS3-2012L, and (g) NICS3-2013.

Selection effect on seed protein content under strategy S1 was assessed and validated using a practical soybean breeding population over two selection cycles. RILs derived from a cross between parents differing in seed protein content were used as the training population, and seed protein content was evaluated across multiple environments from 2009 to 2013 (Supplementary Table 1). In total, 19 QTLs were identified using multi-environment multiple interval mapping (ME-MIM) (Figure 4; Supplementary Table 8). Three major QTLs associated with seed protein content were identified near markers Gm15_04338436S, Gm19_39293939S, and Gm20_44762804S. These three QTLs alleles from the higher seed protein content cultivar “Enrei” revealed an increasing genetic effect on protein content in the seven environments. In contrast, three QTLs (near markers Gm05_28181403S, Gm07_13926582S, and Gm19_00077940S) with increasing genetic effect were identified from the lower seed protein content cultivar “Hyuga.” Previous MARS studies have shown that selection responses increase if relaxed significance levels are used to identify the markers associated with the target traits, and these markers are used as variables in a multiple regression model for selection (Hospital et al., 1997). The markers associated with protein content were independently detected in each phenotypic data set (Figure 4; Supplementary Table 9) using the VBAY model. SNP makers located at Gm15_03756617S were detected in three out of seven phenotype datasets. Four SNP makers (located on Gm15_04338436S, Gm19_38628517S, Gm19_39293939S, and Gm20_45559882S) were detected in two out of the seven phenotype datasets. The remaining SNP markers were detected in one of the seven phenotype datasets. Overall, twenty nine markers were selected as variables for the multiple regression model (“Arrows” in Figure 4; Supplementary Table 2). The effectiveness of marker selection was validated by comparing the accuracy of the prediction model given the 29 selected markers vs. using all available 513 markers (Supplementary Table 10), with a ten-fold cross-validation. We found that when the model was provided with the reduced set of 29 markers, a higher average coefficient value was obtained, indicating that the selected 29 markers were sufficient in predicting protein seed content. The prediction model generated using the 2013 dataset was selected for the following reasons. First, we intended to validate the selection effect in the NICS3 experimental field. The model constructed using the phenotypic data obtained at the same field might reduce the influence of environmental differences on QTL effects detected using ME-MIM, and it is expected to increase the selection accuracy. Second, among the phenotypic datasets obtained at NICS3, the 2012 and 2012 L datasets in 2012 contained excessive missing data, whereas the 2013 data set had no missing data (the column of “Number of Lines” in Supplementary Table 11). Furthermore, the correlation coefficients between the observed values at NICS3 in 2013, 2014, and 2015 and the values estimated by the 2013 prediction model were 0.70, 0.67, and 0.62, respectively (Supplementary Table 10; Supplementary Figure 1), supporting the stability of the prediction model over the years.

### Simulation-Based Selection to Determine the Best Cross Combination Between RILs

**Figure 5:**
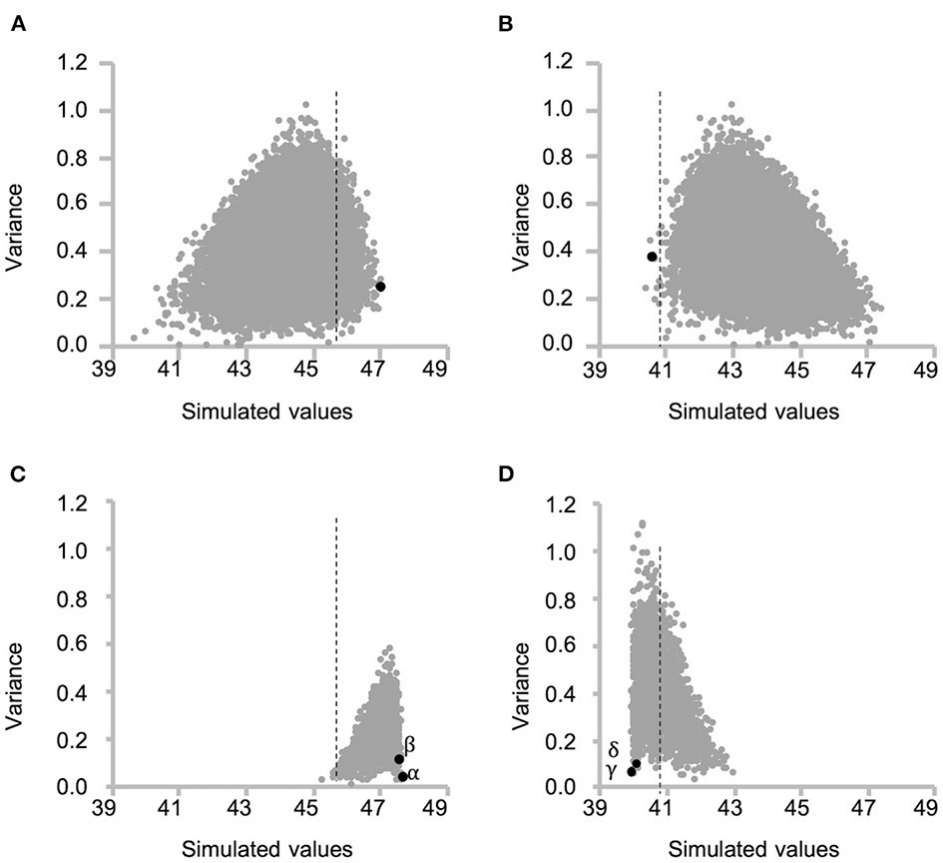
Scatter plot of the mean and variance of simulated protein content of each F_2_ population for all possible cross combinations between recombinant inbred lines (RILs). Each grey dot indicates each cross combination. **(A, C)** and **(B, D)** indicate selection toward higher and lower seed protein content, respectively. **(A)** and (**B**) indicate the variation of the mean of top 5% of F_2_ plants (**A**) and bottom 5% of F_2_ plants (**B**) and variance of F_2_ population for each cross combination at the first selection cycle. The dashed lines indicate the highest **(A, C)** or lowest **(B, D)** estimated value among RILs. Selected best cross combination toward higher [**(A)** RIL048 × RIL176] and lower [**(B)** RIL047 × RIL097] protein content is shown in black dot. **(C, D)** indicate variation of the mean and variance at the second selection cycle from the cross combination of **(A)** and **(B)**, respectively. Greek letters (α−δ) beside the black dots indicate identifiers for the cross combination in the following Figures 6, 7.

Together, the selected 29 marker genotypes of RILs and the 2013 prediction model were used to simulate protein content of the F_2_ progeny (first F_2_) for all the possible 18,721 cross combinations between 194 RILs. The optimal cross for obtaining first F_2_ plants with higher protein content was determined by using the mean simulated values of the top 10 plants (top 5% of progeny) for each cross combination (Figure 5A). Overall, a cross between RIL048 and RIL176 was predicted to yield the highest seed protein content; thus, this cross was carried out to produce first F_2_ population (“black dot” in Figure 5A). For selection toward lower protein content, the mean simulated values for the bottom 10 plants (bottom 5% of progeny) were calculated similarly, leading to the selection of RIL047 and RIL097 for crossing (“black dot” in Figure 5B).

### Comparison Between Simulated and Predicted Values in the First F_2_ Population

**Figure 6:**
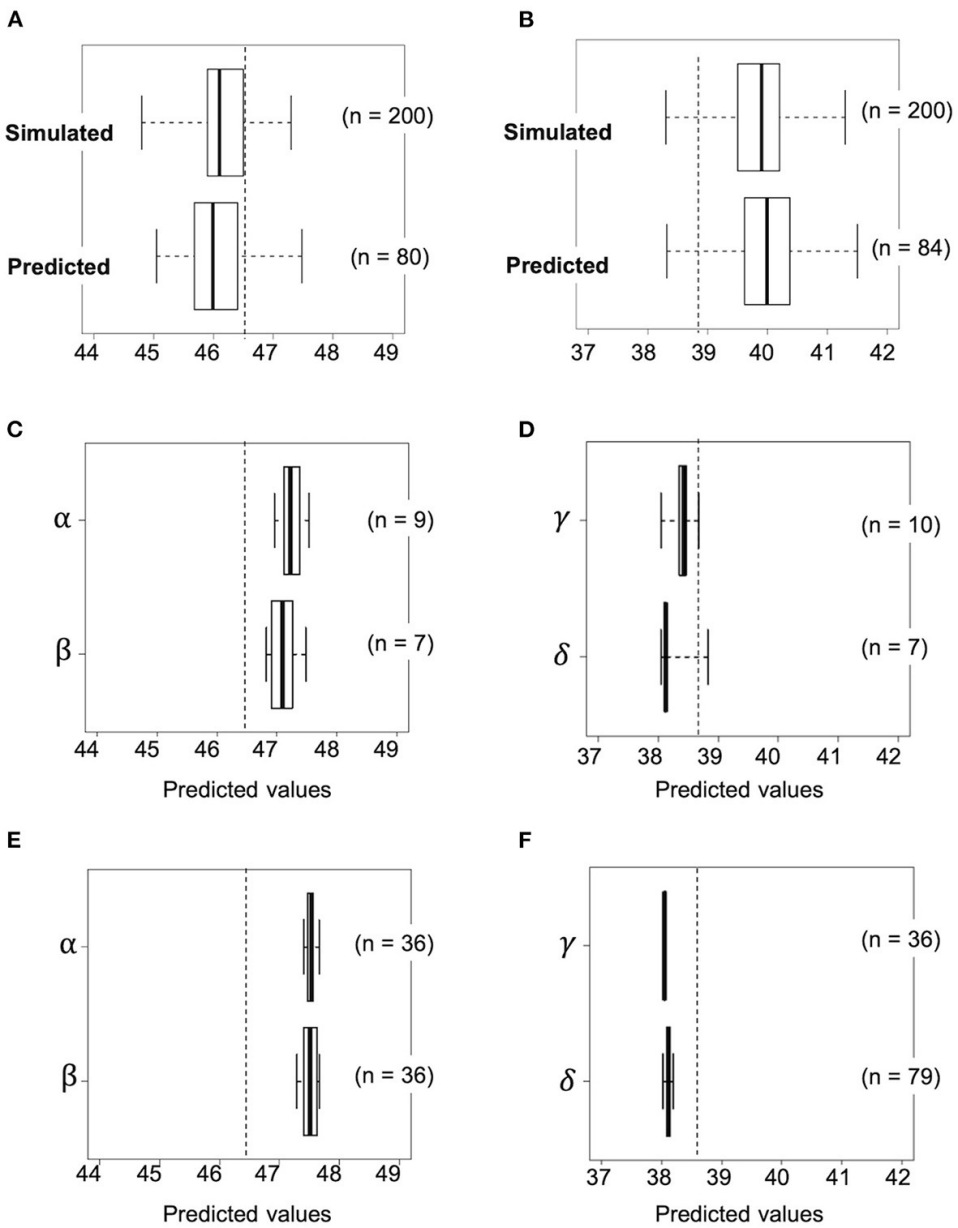
Transition of predicted values based on actual genotypes against selection as the generation progressed. Selection toward high seed protein content by using progeny of a cross combination of RIL048 × RIL176 **(A, C, E)** and low seed protein content by using that of RIL047 × RIL097 **(B, D, F)**. **(A, B)** Upper and lower box plots indicate simulated and predicted values of the F_2_ population at the first selection cycle, respectively. **(C, D)** Boxplots for the predicted values based on the genotype of the second F_1_ plants at the second selection cycle. **(E, F)** Boxplots for the predicted values in the second F_2_ population derived from self-pollination of selected second F_1_ plants. The whiskers of each boxplot indicate the maximum and minimum values. Dashed lines indicate the highest or lowest estimated value among RILs. Greek letters (α−δ) beside the *Y*-axis indicate identifiers for the cross combination.

Accuracy of the simulation data was investigated by calculating the predicted values of the first F_2_ population derived from the selected crosses. This was carried out using the practical marker genotype data for each of the first F_2_ plant and the prediction model. In the first F_2_ population derived from RIL048 × RIL176, the predicted values of several plants were higher than those of line RIL011, which had the highest estimated values of all the RILs (Figure 6A). The frequency of progeny with simulated values exceeding the highest estimated values of RIL011 was 43% (85/200), whereas it was 31% (25/80) when the predicted values of practical progeny were used. The mean predicted values of the top four F_2_ plants (top 5% of the population) were 47.1%, which was similar to the mean simulated values (47.1%) of the top 5% of the population (“black dot” in Figure 5A).

In contrast, the predicted values of several plants in the first F_2_ population derived from the RIL047 × RIL097 cross were lower than those of the RIL with the lowest estimated values (RIL047) (Figure 6B). The frequency of the simulated progeny with simulated values lower than those of RIL047 was 8% (16/200), whereas it was 6% (5/84) compared with the predicted values. The mean predicted values of the lower four F_2_ plants (~lower 5% of the population) were 38.6%, very similar to the mean simulated values (38.6%) of the lower 5%. These results suggest that our simulation method is quite effective in selecting cross combinations.

### Simulation of the Second Cycle and Development of Validation Lines

Progeny with higher or lower protein content than those of the first F_2_ progeny were obtained by simulating the F_2_ population (second F_2_) for all possible cross combinations within each of the first F_2_ population. In this simulation, five F_1_ genotypes were simulated and 50 F_2_ genotypes were then generated by selfing of each F_1_, totaling 250 second F_2_ progeny per cross. A total of 3,160 and 3,845 cross combinations between the first F_2_ plants, derived from RIL048 × RIL176 and RIL047 × RIL097, respectively, were simulated (Figures 5C,D). In the RIL048 × RIL176 second F_2_ population the mean simulated values of the top 12 plants (~top 5% of the population) were used to select the best cross combination for obtaining progeny with higher protein content (Figure 5C). Two individuals with some of the highest mean simulated values were selected for crossing. In contrast, in the RIL047 × RIL097 second F_2_ population the mean simulated values of the bottom 12 plants (~lower 5% of the population) were calculated and used to select cross combinations (Figure 5D). Two individuals with some of the lowest mean simulated protein content were selected for crossing. Each of the second F_1_ plant obtained from each cross combination was grown, and the PV was calculated using practical marker genotypes from each of the second F_1_ plant (Figures 6C,D). One second F_1_ plant from each cross, for the highest or lowest predicted protein content, respectively, was selected from the second F_1_ plants and self-pollinated. The resulting seeds were grown as the second F_2_ population. Marker genotype data obtained from the second F_2_ plants were used to calculate the PVs (Figures 6E,F). The top four plants were selected from each of the second F_2_ population derived from the RIL048 × RIL176 cross, whereas the bottom four plants were selected from each of the second F_2_ population derived from the RIL047 × RIL097 cross. Each selected second F_2_ plant was self-pollinated, and the second F_3_ seeds were used as lines to validate the selection effect.

### Evaluation of Protein Content of the Validation Lines

**Figure 7:**
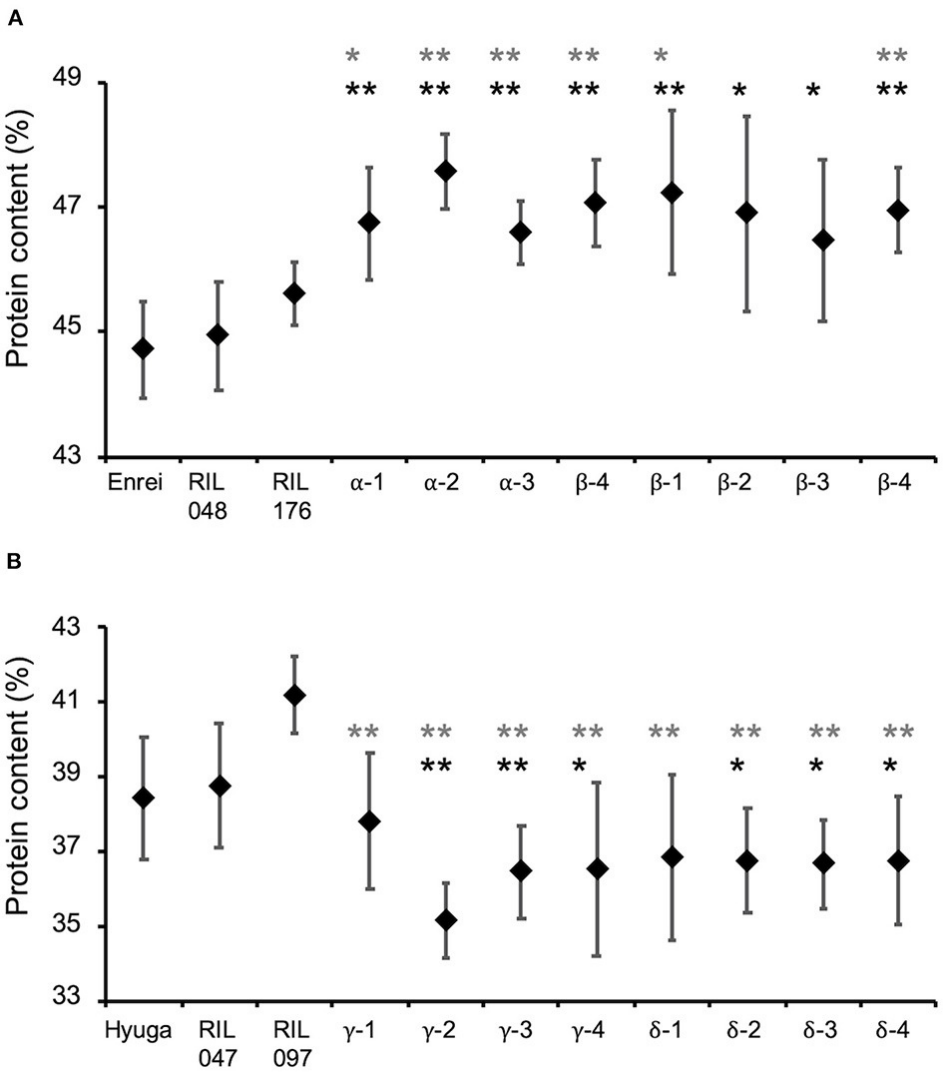
Comparison of protein content of the validation lines with that of parental cultivars and parental recombinant inbred lines (RILs). Selected progeny of RIL048 × RIL176 toward higher protein content **(A)** and that of RIL047 × RIL097 toward lower protein content **(B)**. Greek letters (α−δ) indicate identifiers for the cross combination. The same Greek letter with different numbers indicates lines developed from the same cross combination. Black diamonds and whiskers indicate mean protein content and standard deviation, respectively. Black and grey asterisks indicate the mean protein content of the validation lines were significantly higher than that of their parental lines RIL048 and RIL176 **(A)** or lower than that of RIL047 and RIL097 **(B)** based on one-sided Student's *t*-test, respectively. *, *p* < 0.05; **, *p* < 0.01.

Whether simulation-based selection was able to increase protein content was determined by comparing protein content in eight of the second F_3_ validation lines derived from RIL048 × RIL176, the parents of the RILs, with the estimated and observed top RILs (the protein content of which was the highest among the RILs in 2013–2015) (Supplementary Table 12). The mean protein content of the validation lines was significantly higher than that of the parental RILs (Figure 7A). For instance, the mean protein content of parental RIL048 and RIL176 was 44.9 and 45.6%, respectively, while the mean protein content in the validation lines developed from the second F_2_ population was 46.6–47.6% (“α” in Figure 7A). Similarly, the mean protein content of the validation lines derived from the other second F_2_ population was 46.5–47.2% (“β” in Figure 7A). Among the validation lines, six out of the eight lines showed significantly higher protein content than both parental RILs. Furthermore, the mean protein content of all validation lines was higher than that of the parental cultivar ‘Enrei' and the top RILs (Supplementary Table 12). Similarly, protein content of the validation lines derived from RIL047 × RIL097 (designed for selection toward lower protein content) were compared with those of parental RILs, estimated and observed lowest RILs (the protein content of which was the lowest among the RILs in 2013–2015) (Supplementary Table 13). Mean protein content in all validation lines was lower than that of the parental RILs (Figure 7B). The mean protein content of parental RIL047 and RIL097 was 38.7 and 41.2%, respectively. In contrast, the mean protein contents of the validation lines developed from one second F_2_ population were 35.1–37.8% (“γ” in Figure 7B). Similarly, protein content in the validation lines derived from the other second F_2_ population was 36.7–36.8% (“δ” in Figure 7B). Among the validation lines, six out of the eight lines had significantly lower protein content than the parental RILs. Furthermore, the mean protein content of all validation lines was lower than that of the parental cultivar “Hyuga” and the lowest RILs (Supplementary Table 13). Although significant differences from the parental RILs were not detected in some validation lines, all validation lines were confirmed to be selected for high or low protein content, as expected, and are considered to be superior progeny.

## Discussion

### Efficiency of the Proposed Strategy

The integration of multiple favorable alleles is essential for the improvement of quantitative traits in plant breeding. However, efficient improvement of quantitative traits is not easy, as the probability of obtaining progeny with multiple favorable alleles is considerably low, particularly in self-pollinated crops. Recurrent selection based on genomic information, such as MARS and GS, has been shown to increase the probability of obtaining superior progeny and efficiently improving a target quantitative trait in cross-pollinated crops (Abdulmalik et al., 2017; Crossa et al., 2017; Yabe et al., 2018). However, these methods are not feasible in self-pollinated crops, as obtaining numerous F_1_ hybrids by hand crossing can be quite challenging. Thus, to realistically apply GS and MARS to self-pollinated crops, a strategy for reducing the labor of hand crossing is needed.

To improve the target quantitative traits with minimal hand crossing, we proposed a new strategy, S1, for the selection of specific parental plants expected to produce F_2_ progeny with the best performance from all possible cross combinations in a breeding population based on the computationally simulated phenotypes of the progeny. In the present study, the selection efficiency of S1 was evaluated under several simulation conditions. The MGVs of S1 were significantly higher than those of the single cross strategy based on the PVs of P1 when QTL = 30 and *h*^2^ = 0.6 or 1.0 (Figure 2; Supplementary Tables 3, 4). Moreover, the MGVs of S1 were slightly lower than or similar to those of P5 and P10 during the early cycles (Figure 2; Supplementary Tables 3, 4). The number of cross combinations of S1 to generate the population for the next cycle is one-fifth to one-tenth of that required for P5 and P10. These results suggest that our proposed strategy S1 reduces the number of crosses required; thus, increasing the efficiency of short-term selection.

In contrast, the MGVs of S1 were significantly lower than those of P5 and P10 when QTL = 30 and *h*^2^ = 0.3 (Figure 2; Supplementary Tables 3, 4). No significant differences were observed between S1 and P1 at early selection cycles when QTL = 100 and *h*^2^ = 1.0 (Figure 2; Supplementary Tables 3, 5). Higher selection accuracy was observed for S1 in the first and second cycles when *h*^2^ = 0.6 and 1.0, compared to when *h*^2^ = 0.3 (Supplementary Table 6). Therefore, the simulated results suggest that S1 is efficient for short-term selection when the target trait is governed by a medium number of QTLs and its heritability is high.

### Drawbacks and Future Development of the Simulation-Based Selection Strategy

The strategy S1 has limitations related to the impact of a strong genetic bottleneck during selection. The proportion of unfixed QTL in S1 rapidly declined during the first cycle, whereas those strategies based on multiple crosses (S5, S10, P5, and P10) maintained higher values (Figure 3; Supplementary Table 7). This suggests that a strong genetic bottleneck due to the limited number of crosses causes a rapid decline in genetic variation, particularly in the first cycle. Genetic variation is the source of improvement in the next generation; thus, maintaining genetic variation with less fixation of unfavorable alleles is important toward obtaining more genetic gains in the following cycles. Daetwyler et al. (2015) proposed the optimal haploid value (OHV), which calculates the potential value of a plant when the best completely homozygous progeny (such as doubled haploids) are generated from each plant. The genetic variance of OHV selection was higher than that of selection based on genomic estimated breeding value (i.e., GS), and more genetic gain was obtained, particularly in the later cycles (Daetwyler et al., 2015). Thus, it may be beneficial to apply S1 to obtain more genetic gain. Alternatively, the multiple cross combination strategies, S5 and S10, were effective in maintaining genetic variation (Figure 3; Supplementary Table 7) to obtain more genetic gain (Figure 2; Supplementary Table 3). Previously, several selection strategies assuming multiple cross combinations, such as genotype building selection (GB, Kemper et al., 2012) and optimal population value (OPV, Goiffon et al., 2017) have been proposed to select a set of plants that are more likely to produce superior progeny when crossed with each other. These are superior to GS in maintaining high genetic variance over selection cycles (Goiffon et al., 2017). Although the difficulty in producing the next generation in multiple cross combinations remains a major issue in many self-pollinated crops, these selection methods would improve genetic gain more in the later selection cycles.

### Prospects for Further Improvement of Seed Protein Content in Soybean

We chose seed protein content as the quantitative trait manipulated in this study, as this trait is agriculturally relevant, both for livestock feed and human consumption. In particular, in Japan, high seed protein content is important for developing new cultivars of soybean, as this trait is known to be positively correlated with the consistency of tofu (Toda et al., 2003), a healthy and traditional soy-based food. Over the past two decades, more than 160 QTLs have been reported as associated with seed protein content in soybean (Patil et al., 2017). The Soybean Genetics Committee officially designated two stable seed protein content-related QTLs on chromosome 15 and 20 as reliable for marker-assisted selection. In the present study, a QTL on chromosome 15 with stable effects was detected in a similar genomic region (Figure 4; Supplementary Table 9). In contrast, other DNA markers included as variables in the prediction model revealed low genetic effects depending on the environment (i.e., year, location, and field type) (Supplementary Table 11). Previous studies have shown that temperature during the pod maturation stage influences seed protein content (Patil et al., 2017). Environmental influence must be considered when developing a general selection model. Recently, some studies have proposed that the performance of plants cultivated under various environmental conditions was predicted by the integration of a crop model into GS utilizing environmental information such as temperature, photoperiod, precipitation, and sowing date (Heslot et al., 2014; Technow et al., 2015; Onogi et al., 2016). These integrated models are more accurate than the models constructed using only genome-wide DNA marker information when phenotypic datasets from multienvironmental conditions are available. Future integration of such models with our proposed strategy would improve the development of plants with stable agronomic performance across different environmental conditions.

## Data Availability Statement

The original contributions generated for the study are included in the article/Supplementary Materials, further inquiries can be directed to the corresponding author/s.

## Author Contributions

DS, MT, and AK designed the study and analyzed the data. DS, MT, MS, TY, and AK collected the phenotype data. DS, TS, and KM collected the genotype data. DS, SY, and HI developed the simulation program. DS, MT, MS, TY, MI, and AK drafted the manuscript. All authors contributed to the article and approved the submitted version.

## Conflict of Interest

The authors declare that the research was conducted in the absence of any commercial or financial relationships that could be construed as a potential conflict of interest.

## Publisher's Note

All claims expressed in this article are solely those of the authors and do not necessarily represent those of their affiliated organizations, or those of the publisher, the editors and the reviewers. Any product that may be evaluated in this article, or claim that may be made by its manufacturer, is not guaranteed or endorsed by the publisher.

## Abbreviations

GS: genomic selection
GV: genetic value
LASSO: least absolute shrinkage and selection operator
MARS: marker-assisted recurrent selection
ME-MIM: multi-environment multiple interval mapping
MGV: maximum genetic value
NICS: experimental field name
P1: conventional recurrent selection strategy with single cross
P5 and P10: conventional recurrent selection strategy with multiple crosses
QTL: quantitative trait loci
RIL: recombinant inbred line
S1: proposed selection strategy with single cross
S5 and S10: proposed selection strategy with multiple crosses
SNP: single nucleotide polymorphism
VBAY: variational method for Bayesian hierarchical regression.

## References

[B1] Abdulmalik R. O. Menkir A. Meseka S. K. Unachukwu N. Ado S. G. Olarewaju J. D.. (2017). Genetic gains in grain yield of a maize population improved through marker assisted recurrent selection under stress and non-stress conditions in West Africa. Front. Plant Sci. 8:841. 10.3389/fpls.2017.00841 28588598 PMC5438988

[B2] Bernardo R. (2008). Molecular markers and selection for complex traits in plants: learning from the last 20 years. Crop Sci. 48, 1649–1664. 10.2135/cropsci2008.03.0131

[B3] Bernardo R. (2010). Genomewide selection with minimal crossing in self-pollinated crops. Crop Sci. 50, 624–627. 10.2135/cropsci2009.05.0250

[B4] Beyene Y. Semagn K. Crossa J. Mugo S. Atlin G. N. Tarekegne A.. (2016). Improving maize grain yield under drought stress and non-stress environments in sub-saharan africa using marker-assisted recurrent selection. Crop Sci. 56, 344–353. 10.2135/cropsci2015.02.0135

[B5] Beyene Y. Semagn K. Mugo S. Tarekegne A. Babu R. Meisel B.. (2015). Genetic gains in grain yield through genomic selection in eight bi-parental maize populations under drought stress. Crop Sci. 55, 154–163. 10.2135/cropsci2014.07.0460

[B6] Bonferroni C. E. (1936). Teoria statistica delle classi e calcolo delle probabilità. Pubblicazioni del R Istituto Superiore di Scienze Economiche e Commerciali di Firenze 8, 3–62.

[B7] Bos I. Caligari P. (2008). Selection Methods in Plant Breeding. 2nd ed. Dordrecht: Springer. 10.1007/978-1-4020-6370-1

[B8] Brown J. Caligari P. (2008). An Introduction to Plant Breeding. Hoboken, NJ: Blackwell Publishing Ltd. 10.1002/9781118685228

[B9] Candwell B. E. (1973). SOYBEANS: Improvement, Production, and Uses. Wisconsin: American Society of Agronomy and Crop Science Society of America, Madison

[B10] Churchill G. A. Doerge R. W. (1994). Empirical threshold values for quantitative trait mapping. Genetics 138, 963–971. 10.1093/genetics/138.3.963 7851788 PMC1206241

[B11] Crossa J. Perez-Rodriguez P. Cuevas J. Montesinos-Lopez O. Jarquin D. de Los Campos G.. (2017). Genomic selection in plant breeding: methods, models, and perspectives. Trends Plant Sci. 22, 961–975. 10.1016/j.tplants.2017.08.011 28965742

[B12] Daetwyler H. D. Hayden M. J. Spangenberg G. C. Hayes B. J. (2015). Selection on optimal haploid value increases genetic gain and preserves more genetic diversity relative to genomic selection. Genetics 200, 1341–1348. 10.1534/genetics.115.178038 26092719 PMC4574260

[B13] Eathington S. R. Crosbiea T. M. Edwardsb M. D. Reiterc R. S. Bullc J. K. (2007). Molecular markers in a commercial breeding program. Crop Sci. 47(Supplement_3), S154–S163. 10.2135/cropsci2007.04.0015IPBS

[B14] FAO (2015). FAO Statistical Pocketbook. Geneva: FAO

[B15] Friedman J. Hastie T. Tibshirani R. (2010). Regularization paths for generalized linear models via coordinate descent. J. Stat. Softw. 33, 1–22. 10.18637/jss.v033.i01 20808728 PMC2929880

[B16] Goiffon M. Kusmec A. Wang L. Hu G. Schnable P. S. (2017). Improving response in genomic selection with a population-based selection strategy: optimal population value selection. Genetics 206, 1675–1682. 10.1534/genetics.116.197103 28526698 PMC5500159

[B17] Heslot N. Akdemir D. Sorrells M. E. Jannink J. L. (2014). Integrating environmental covariates and crop modeling into the genomic selection framework to predict genotype by environment interactions. Theor. Appl. Genet. 127, 463–480. 10.1007/s00122-013-2231-5 24264761

[B18] Hoffman G. E. Logsdon B. A. Mezey J. G. (2013). PUMA: a unified framework for penalized multiple regression analysis of GWAS data. PLoS Comput. Biol. 9:e1003101. 10.1371/journal.pcbi.1003101 23825936 PMC3694815

[B19] Hospital F. Moreau L. Lacoudre F. Charcosset A. Gallais A. (1997). More on the efficiency of marker-assisted selection. Theor. Appl. Genetics 95, 1181–1189. 10.1007/s001220050679

[B20] Iwata H. Hayashi T. Terakami S. Takada N. Saito T. Yamamoto T. (2013). Genomic prediction of trait segregation in a progeny population: a case study of Japanese pear (*Pyrus pyrifolia*). BMC Genet. 14:81. 10.1186/1471-2156-14-81 24028660 PMC3847345

[B21] John M. P. (1987). Breeding Field Crops Third Edition. An avi Book. New York, NY: Van Nostrand Reinhold.

[B22] Kemper K. E. Bowman P. J. Pryce J. E. Hayes B. J. Goddard M. E. (2012). Long-term selection strategies for complex traits using high-density genetic markers. J. Dairy Sci. 95, 4646–4656. 10.3168/jds.2011-5289 22818479

[B23] Khosla S. Augustus M. Brahmachari V. (1999). Sex-specific organisation of middle repetitive DNA sequences in the mealybug *Planococcus lilacinus*. Nucleic Acids Res. 27, 3745–3751. 10.1093/nar/27.18.3745 10471745 PMC148631

[B24] Korol A. B. Ronin Y. I. Itskovich A. M. Peng J. Nevo E. (2001). Enhanced efficiency of quantitative trait loci mapping analysis based on multivariate complexes of quantitative traits. Genetics 157, 1789–1803. 10.1093/genetics/157.4.1789 11290731 PMC1461583

[B25] Korol A. B. Ronin Y. I. Nevo E. (1998). Approximate analysis of QTL-environment interaction with no limits on the number of environments. Genetics 148, 2015–2028. 10.1093/genetics/148.4.2015 9560414 PMC1460115

[B26] Lande R. Thompson R. (1990). Efficiency of marker-assisted selection in the improvement of quantitative traits. Genetics 124, 743–756. 10.1093/genetics/124.3.743 1968875 PMC1203965

[B27] Lehermeier C. Teyssèdre S. Schön C. C. (2017). Genetic gain increases by applying the usefulness criterion with improved variance prediction in selection of crosses. Genetics 207, 1651–1661. 10.1534/genetics.117.300403 29038144 PMC5714471

[B28] Logsdon B. A. Hoffman G. E. Mezey J. G. (2010). A variational Bayes algorithm for fast and accurate multiple locus genome-wide association analysis. BMC Bioinform. 11:58. 10.1186/1471-2105-11-58 20105321 PMC2824680

[B29] Massman J. M. Jung H. J. G. Bernardo R. (2013). Genomewide selection versus marker-assisted recurrent selection to improve grain yield and stover-quality traits for cellulosic ethanol in maize. Crop Sci. 53, 58–66. 10.2135/cropsci2012.02.0112

[B30] Meuwissen T. H. Hayes B. J. Goddard M. E. (2001). Prediction of total genetic value using genome-wide dense marker maps. Genetics 157, 1819–1829. 10.1093/genetics/157.4.1819 11290733 PMC1461589

[B31] Mohsen M. Tyler T. Kevin P. S. (2015). PopVar: A genome-wide procedure for predicting genetic variance and correlated response in biparental breeding populations. Crop Sci. 55, 2068–2077. 10.2135/cropsci2015.01.0030

[B32] Oeth P. del Mistro G. Marnellos G. Shi T. van den Boom D. (2009). Qualitative and quantitative genotyping using single base primer extension coupled with matrix-assisted laser desorption/ionization time-of-flight mass spectrometry (MassARRAY). Methods Mol. Biol. 578, 307–343. 10.1007/978-1-60327-411-1_20 19768603

[B33] Onogi A. Watanabe M. Mochizuki T. Hayashi T. Nakagawa H. Hasegawa T.. (2016). Toward integration of genomic selection with crop modelling: the development of an integrated approach to predicting rice heading dates. Theor. Appl. Genet. 129, 805–817. 10.1007/s00122-016-2667-5 26791836

[B34] Patil G. Mian R. Vuong T. Pantalone V. Song Q. Chen P.. (2017). Molecular mapping and genomics of soybean seed protein: a review and perspective for the future. Theor. Appl. Genet. 130, 1975–1991. 10.1007/s00122-017-2955-8 28801731 PMC5606949

[B35] R Core Team (2018) A Language and Environment for Statistical Computing. R Foundation for Statistical Computing Vienna, Austria. Available online at: https://www.r-project.org (accessed August 16, 2021).

[B36] Schnell F. W. Utz H. F. (1975). F1-Leistung und Elternwahl in der Züchtung von Selbstbefruchtern. Gumpenstein: BAL Gumpenstein, 234–258.

[B37] Technow F. Messina C. D. Totir L. R. Cooper M. (2015). Integrating crop growth models with whole genome prediction through approximate bayesian computation. PLoS ONE 10:e0130855. 10.1371/journal.pone.0130855 26121133 PMC4488317

[B38] Tester M. Langridge P. (2010). Breeding technologies to increase crop production in a changing world. Science 327, 818–822. 10.1126/science.1183700 20150489

[B39] Toda K. Ono T. Kitamura K. Hajika M. Takahashi K. Nakamura Y. (2003). Seed protein content and consistency of tofu prepared with different magnesium chloride concentrations in six Japanese soybean varieties. Breed. Sci. 53, 217–223. 10.1270/jsbbs.53.217

[B40] Van Ooijen J. W. Voorrips R. E. (2001). JoinMap® Version 4.0: Software for the Calculation of Genetic Linkage Maps. Wageningen: Plant Research International.

[B41] Yabe S. Hara T. Ueno M. Enoki H. Kimura T. Nishimura S.. (2018). Potential of genomic selection in mass selection breeding of an allogamous crop: an empirical study to increase yield of common buckwheat. Front. Plant Sci. 9:276. 10.3389/fpls.2018.00276 29619035 PMC5871932

[B42] Yabe S. Iwata I. Jannink J. (2017). A simple package to script and simulate breeding schemes: the breeding scheme language. Crop Sci. 57, 1347–1354. 10.2135/cropsci2016.06.0538

[B43] Zhong S. Jannink J. L. (2007). Using quantitative trait loci results to discriminate among crosses on the basis of their progeny mean and variance. Genetics 177, 567–576. 10.1534/genetics.107.075358 17660556 PMC2013701

