# Supplementary material for: Improving Quantitative Traits in Self-Pollinated Crops Using Simulation-Based Selection With Minimal Crossing
Source: Front Plant Sci. 2021 Sep 1;12:729645. doi: 10.3389/fpls.2021.729645 (PMC8443513; doi:10.3389/fpls.2021.729645)
Supplement: Supplementary file 2 [file Presentation_1.PPTX]

## Slide 1
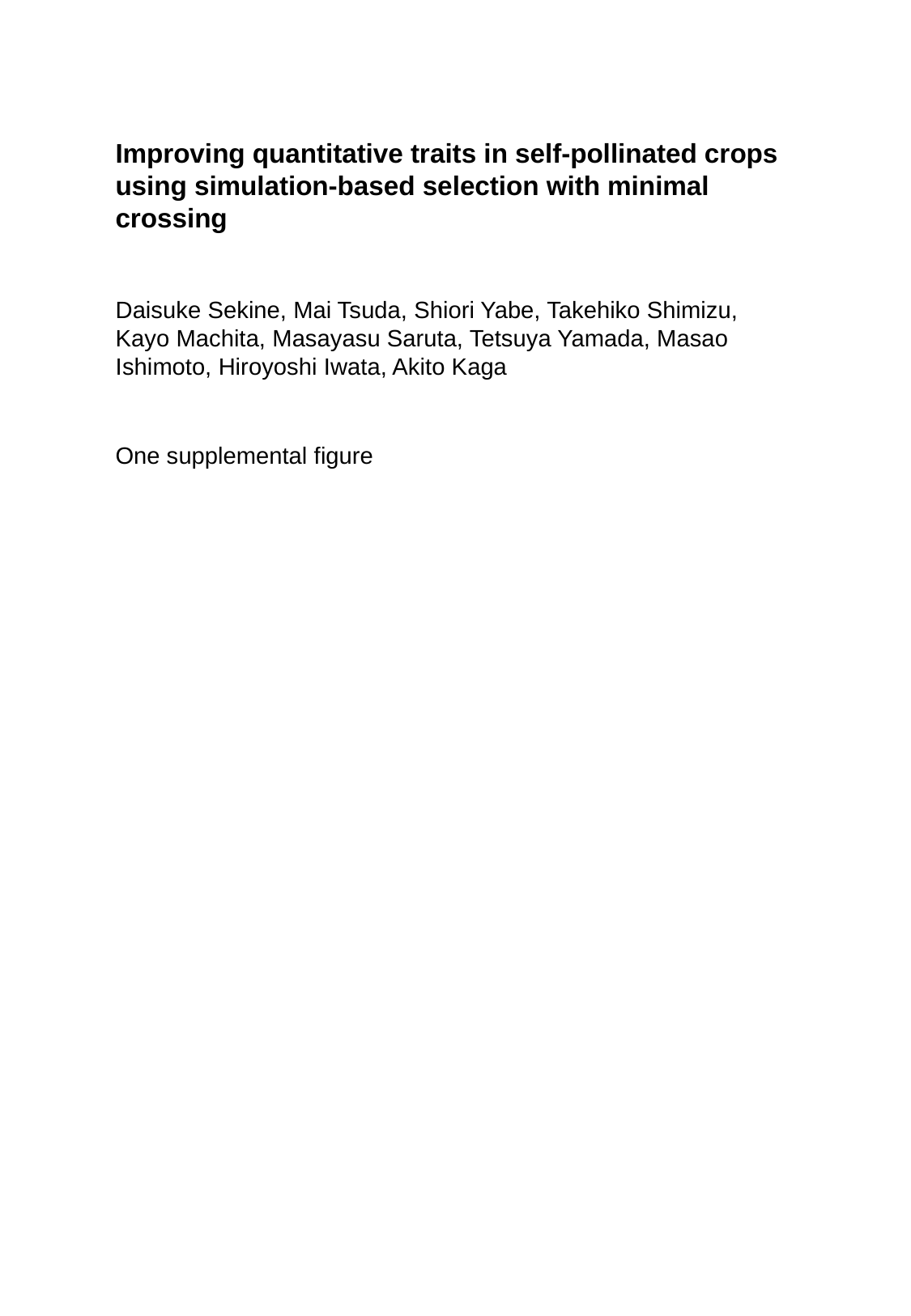

Improving quantitative traits in self-pollinated crops using simulation-based selection with minimal crossing
Daisuke Sekine, Mai Tsuda, Shiori Yabe, Takehiko Shimizu, Kayo Machita, Masayasu Saruta, Tetsuya Yamada, Masao Ishimoto, Hiroyoshi Iwata, Akito Kaga
One supplemental figure

## Slide 2
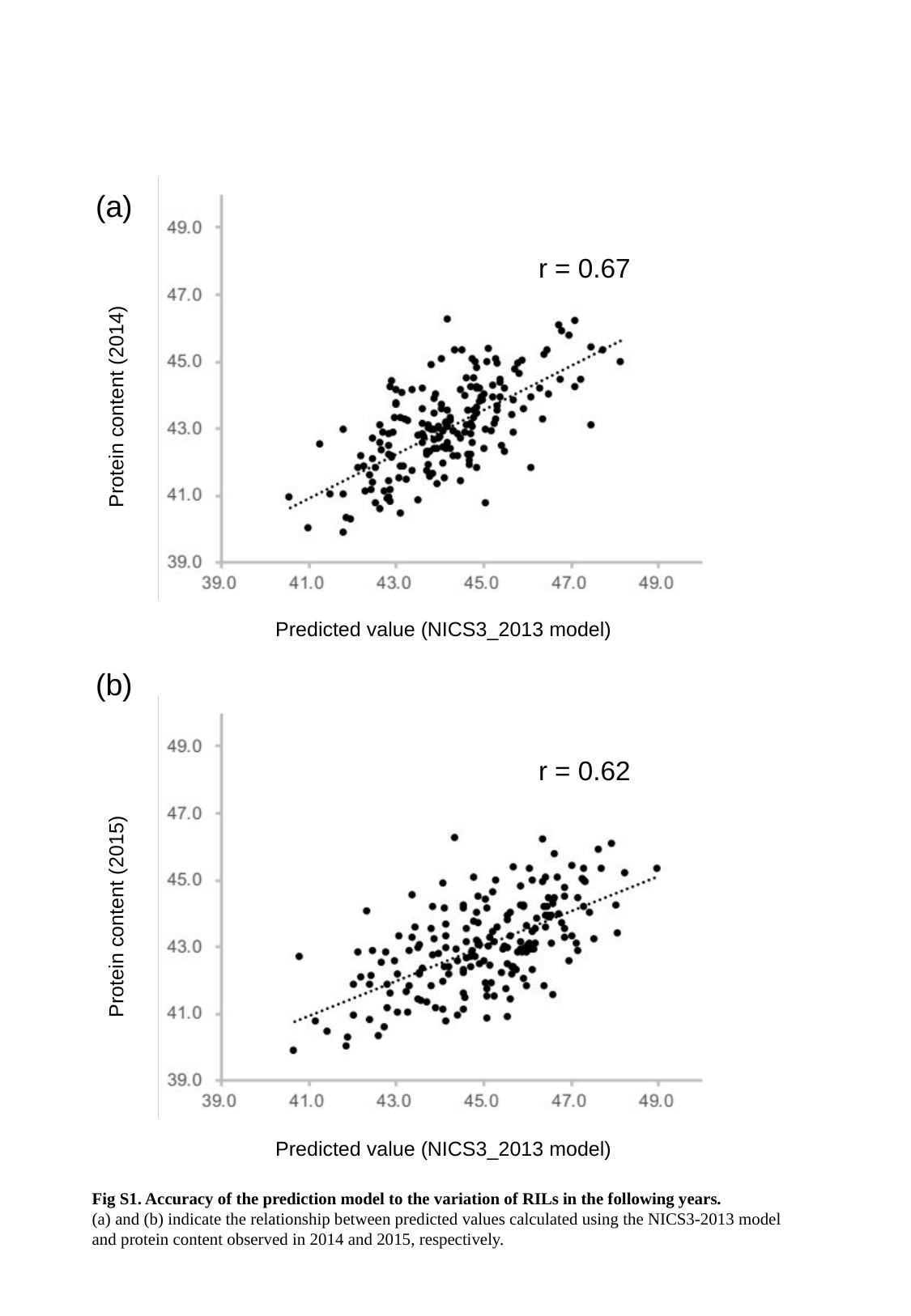

(a)
r = 0.67
Protein content (2014)
Predicted value (NICS3_2013 model)
(b)
r = 0.62
Protein content (2015)
Predicted value (NICS3_2013 model)
Fig S1. Accuracy of the prediction model to the variation of RILs in the following years.
(a) and (b) indicate the relationship between predicted values calculated using the NICS3-2013 model and protein content observed in 2014 and 2015, respectively.
